# Optimization of papain-assisted hydrolysis for salmon by-product protein powder production using response surface methodology

**DOI:** 10.1038/s41598-026-52153-y

**Published:** 2026-05-07

**Authors:** Tanakorn Rachapila

**Affiliations:** Faculty of Agricultural Technology, Sakon Nakhon Rajabhat University, Sakon Nakhon, 47000 Thailand

**Keywords:** Salmon by-products, Papain, Enzymatic hydrolysis, Response surface methodology, Protein powder, Amino acid composition, Biochemistry, Biological techniques, Biotechnology, Chemistry

## Abstract

This study optimized papain-assisted enzymatic hydrolysis of salmon processing by-products using response surface methodology. A central composite design with three factorspapain concentration (1,591–18,409 U/g substrate protein), reaction time (99.5–200.5 min), and temperature (43.2–76.8 °C)was employed to optimize degree of hydrolysis (DH), protein recovery (PR), peptide yield (PY), and protein solubility (PS). Quadratic models demonstrated excellent predictive capability (R² > 0.98). Papain concentration exhibited the most significant positive effect on all responses (*P* < 0.001). Optimal conditions were: papain concentration 15,000 U/g substrate protein, reaction time 165 min, and temperature 55 °C, yielding **predicted** DH 15.42%, PR 86.85%, PY 68.92%, and PS 77.64%, which were subsequently validated by triplicate confirmation experiments (relative errors 0.9–1.6%). The spray-dried powder (with 15% w/w maltodextrin as carrier) contained 75.30% protein, 3.57% fat, 3.51% ash, 2.24% moisture, and 15.38% carbohydrate (by difference). Essential amino acids accounted for 46.71 g/100 g protein (45.26% of total amino acids), exceeding the FAO (2013) reference pattern. Lysine (8.95 g/100 g protein) and leucine (8.24 g/100 g protein) were the predominant essential amino acids. The powder exhibited excellent solubility (> 85% at pH 3–9) and favorable emulsifying properties (EAI 45.8 m²/g protein, ESI 42.5 min). These findings demonstrate that papain-assisted hydrolysis effectively converts salmon by-products into functional protein ingredients for food applications.

## Introduction

The global salmon industry generates substantial quantities of processing by-products, including heads, frames, skin, viscera, and trimmings, accounting for 30–50% of the whole fish mass^1,2^. Traditionally, these by-products have been underutilized or directed to low-value applications such as animal feed or disposal, resulting in economic losses and environmental concerns^3^. In the context of circular economy and sustainable food systems, valorization of fish processing by-products into high-value functional ingredients has attracted increasing attention^4,5^.

Salmon by-products are rich in high-quality proteins, typically containing 15–20% protein on a wet-weight basis, with all essential amino acids, bioactive peptides with various health-promoting properties, and lipids rich in long-chain n-3 polyunsaturated fatty acids^6,7^. Among protein modification methods including chemical hydrolysis (acid or alkali), thermal processing, and fermentation enzymatic hydrolysis is widely recognized as the most suitable technique for converting fish proteins into peptides and protein hydrolysates with improved solubility, digestibility, and bioactivities, because it operates under mild pH and temperature conditions, preserves the nutritional quality of amino acids, and allows precise control of the degree of hydrolysis without generating toxic by-products associated with chemical treatments^8,9^. Among proteolytic enzymes, papain a cysteine protease derived from Carica papaya latex is particularly attractive for hydrolyzing fish muscle and collagenous proteins due to its broad substrate specificity that enables cleavage at multiple sites along the polypeptide chain, its high activity over a wide pH range (5.0–8.0), relatively low cost compared to commercial microbial proteases, and Generally Recognized as Safe (GRAS) status in food applications^10,11^.

Previous studies have demonstrated that enzymatic hydrolysis of fish by-products can produce protein hydrolysates with enhanced functional properties including solubility, emulsifying and foaming capacities, making them suitable as ingredients in beverages, emulsified products, and nutraceuticals^12–14^. Idowu et al.^15^ reported that hydrolysates from salmon frames using Alcalase and papain at 3% enzyme concentration for 180 min yielded approximately 24–26% solids containing 79–82% protein. Gbogouri et al.^16^ demonstrated that, using Alcalase 2.4 L at 0.5–2.0% (v/w) concentration, increasing degree of hydrolysis (DH) from 11.5% to 17.3% markedly improved protein solubility while lower DH favored emulsifying capacity and stability.

Response surface methodology (RSM) is a powerful statistical technique for optimizing complex processes involving multiple variables and their interactions^17^. Central composite design (CCD) is particularly useful for fitting second-order polynomial models and identifying optimal conditions through minimal experimental runs^18^. Several studies have successfully applied RSM to optimize enzymatic hydrolysis of fish proteins, demonstrating its effectiveness in maximizing protein recovery and functional properties^19,20^.

Despite considerable research on fish protein hydrolysates, systematic optimization of papain-assisted hydrolysis of salmon by-products using RSM, combined with comprehensive characterization of the resulting spray-dried protein powder including detailed amino acid profiling, remains limited. Therefore, the objectives of this study were: (i) to optimize papain hydrolysis conditions (enzyme concentration, reaction time, and temperature) using RSM based on degree of hydrolysis, protein recovery, peptide yield, and protein solubility; (ii) to produce stable salmon protein powder from the optimized hydrolysate by spray-drying; and (iii) to characterize the proximate composition, amino acid profile, and techno-functional properties of the resulting powder.

## Results

### Model fitting and statistical analysis

The experimental design matrix with observed and model-predicted response values is presented in Table 1. The degree of hydrolysis ranged from 3.12% to 15.85%, protein recovery from 61.31% to 87.18%, peptide yield from 15.00% to 70.27%, and protein solubility from 45.91% to 82.15% across the 20 experimental runs. Predicted values calculated from the fitted quadratic equations (Table 2) closely matched the observed values for all runs, supporting the high R² values (> 0.98) obtained for all response models. The wide range of responses indicated that the selected factor levels adequately captured the experimental domain for optimization.

**Table 1:** Central composite design matrix with experimental conditions, observed values, and model-predicted values for 20 runs.

Run	Type	X₁	X₂	X₃	Enzyme (U/g substrate protein)	Time (min)	Temp (°C)	DH obs (%)	DH pred (%)	PR obs (%)	PR pred (%)	PY obs (%)	PY pred (%)	PS obs (%)	PS pred (%)
1.00	Factorial	−1	−1	−1	5000	120	50	5.29	5.75	67.30	66.63	25.98	26.40	55.26	55.14
2.00	Factorial	1	−1	−1	15,000	120	50	14.35	13.95	82.10	81.34	57.95	58.81	76.85	76.23
3.00	Factorial	−1	1	−1	5000	180	50	6.33	6.75	70.24	69.79	32.05	31.86	56.66	56.04
4.00	Factorial	1	1	−1	15,000	180	50	15.12	15.43	87.18	86.70	69.47	69.82	77.87	78.54
5.00	Factorial	−1	−1	1	5000	120	70	4.14	4.05	67.12	66.36	23.19	23.89	49.49	49.74
6.00	Factorial	1	−1	1	15,000	120	70	12.34	12.13	80.46	79.67	49.02	50.26	67.67	69.22
7.00	Factorial	−1	1	1	5000	180	70	5.01	5.63	69.26	68.77	29.53	29.72	53.18	54.72
8.00	Factorial	1	1	1	15,000	180	70	14.51	14.19	84.84	84.27	61.01	61.64	74.57	75.61
9.00	Axial	−1.682	0	0	1591	150	60	3.12	2.23	61.31	62.12	15.00	14.84	45.91	45.73
10.00	Axial	1.682	0	0	18,409	150	60	15.85	16.32	86.58	87.53	70.27	68.95	82.15	81.03
11.00	Axial	0	−1.682	0	10,000	99.5	60	8.77	8.98	71.72	72.89	42.63	41.23	62.48	62.30
12.00	Axial	0	1.682	0	10,000	200.5	60	12.06	11.55	78.84	79.42	55.47	55.39	69.55	68.43
13.00	Axial	0	0	−1.682	10,000	150	43.2	11.70	11.29	76.41	77.24	48.28	47.94	68.04	68.90
14.00	Axial	0	0	1.682	10,000	150	76.8	8.72	8.82	74.02	74.97	40.09	38.95	64.06	61.90
15.00	Center	0	0	0	10,000	150	60	12.24	12.04	79.90	79.91	52.80	52.83	68.69	69.80
16.00	Center	0	0	0	10,000	150	60	12.54	12.04	79.91	79.91	52.66	52.83	68.37	69.80
17.00	Center	0	0	0	10,000	150	60	11.66	12.04	79.07	79.91	53.42	52.83	69.80	69.80
18.00	Center	0	0	0	10,000	150	60	12.42	12.04	79.63	79.91	52.00	52.83	70.77	69.80
19.00	Center	0	0	0	10,000	150	60	11.55	12.04	81.09	79.91	53.90	52.83	70.98	69.80
20.00	Center	0	0	0	10,000	150	60	11.71	12.04	80.13	79.91	51.96	52.83	69.98	69.80

Enzyme concentrations are expressed in U/g substrate protein. ‘obs’ = observed experimental value; ‘pred’ = model-predicted value calculated from the fitted quadratic regression equations in Table 2.

**Table 2:** Regression coefficients (coded units) for the fitted quadratic models.

Term	DH Coef	DH *P*	PR Coef	PR *P*	PY Coef	PY *P*	PS Coef	PS *P*
Intercept (β₀)	12.044	< 0.001	79.905	< 0.001	52.832	< 0.001	69.802	< 0.001
X₁ (β₁)	4.191	< 0.001	7.553	< 0.001	16.083	< 0.001	10.494	< 0.001
X₂ (β₂)	0.766	< 0.001	1.941	< 0.001	4.211	< 0.001	1.823	< 0.001
X₃ (β₃)	−0.734	< 0.001	−0.674	0.034	−2.671	< 0.001	−2.081	< 0.001
X₁² (β₁₁)	−0.979	< 0.001	−1.796	< 0.001	−3.866	< 0.001	−2.271	< 0.001
X₂² (β₂₂)	−0.629	0.002	−1.325	< 0.001	−1.599	< 0.001	−1.569	0.002
X₃² (β₃₃)	−0.702	0.001	−1.342	< 0.001	−3.318	< 0.001	−1.557	0.002
X₁X₂ (β₁₂)	0.119	0.581	0.548	0.157	1.388	0.005	0.354	0.504
X₁X₃ (β₁₃)	−0.029	0.893	−0.353	0.348	−1.510	0.003	−0.404	0.447
X₂X₃ (β₂₃)	0.144	0.505	−0.188	0.612	0.093	0.813	1.021	0.073

Coef = regression coefficient. P = P-value. Bold terms are significant at *P* < 0.05.

The ANOVA results for the fitted quadratic models are summarized in Table 3. All four models were highly significant (*p* < 0.0001) with non-significant lack-of-fit tests (*p* > 0.05), indicating adequate model fit. The coefficient of determination (R²) values were 0.988, 0.989, 0.997, and 0.988 for DH, PR, PY, and PS, respectively. The adjusted R² values (0.977, 0.979, 0.995, and 0.978) were in reasonable agreement with the predicted R² values (0.921, 0.931, 0.984, and 0.929 for DH, PR, PY, and PS, respectively), with differences less than 0.07, confirming the models’ reliability for prediction within the experimental domain. The lack-of-fit tests were non-significant for all models (*p* = 0.135, 0.090, 0.131, and 0.151 for DH, PR, PY, and PS, respectively), further confirming adequate model fit without overfitting.

**Table 3:** ANOVA summary for response surface quadratic models.

Response	*R*²	Adj. *R*²	Pred. *R*²	F-value	Model *p*-value	Lack-of-Fit *p*-value	RMSE	CV (%)
DH (%)	0.988	0.977	0.921	89.19	< 0.0001***	0.135	0.59	5.62
PR (%)	0.989	0.979	0.931	99.87	< 0.0001***	0.090	1.01	1.32
PY (%)	0.997	0.995	0.984	405.77	< 0.0001***	0.131	1.08	2.31
PS (%)	0.988	0.978	0.929	93.00	< 0.0001***	0.151	1.44	2.18

*** *p* < 0.001; RMSE = Root mean square error; CV = Coefficient of variation; Pred. R² = Predicted R²; Lack-of-Fit p-values > 0.05 indicate adequate model fit.

### Effect of process variables on responses

The regression coefficients for all response models are presented in Table 2. Papain concentration (X₁) exhibited the most significant positive effect on all responses (*p* < 0.001), with linear coefficients of 4.19, 7.55, 16.08, and 10.49 for DH, PR, PY, and PS, respectively. This finding is consistent with enzyme kinetics principles, where higher enzyme concentration increases the rate of peptide bond cleavage until substrate saturation occurs. The substantial effect of papain concentration on protein recovery (coefficient = 7.55) indicates that enzyme dosage is critical for maximizing the extraction of soluble proteins from salmon by-products.

Reaction time (X₂) also showed significant positive effects on all responses (*p* < 0.01), with coefficients ranging from 0.77 (DH) to 4.21 (PY). Extended hydrolysis time allowed for more complete protein breakdown and peptide release, contributing to improved yields. In contrast, reaction temperature (X₃) exhibited significant negative effects on DH, PY, and PS (*p* < 0.01), with coefficients of − 0.73, − 2.67, and − 2.08, respectively. This negative relationship suggests that temperatures above the optimal range (55–60 °C) led to enzyme denaturation and reduced hydrolytic efficiency.

All quadratic terms (X₁², X₂², X₃²) were significant and negative, indicating the presence of optimum conditions within the experimental domain rather than monotonic relationships. The negative quadratic coefficients confirm that the response surfaces exhibit maximum points, validating the appropriateness of CCD for optimization.

### Response surface analysis

**Fig. 1:**
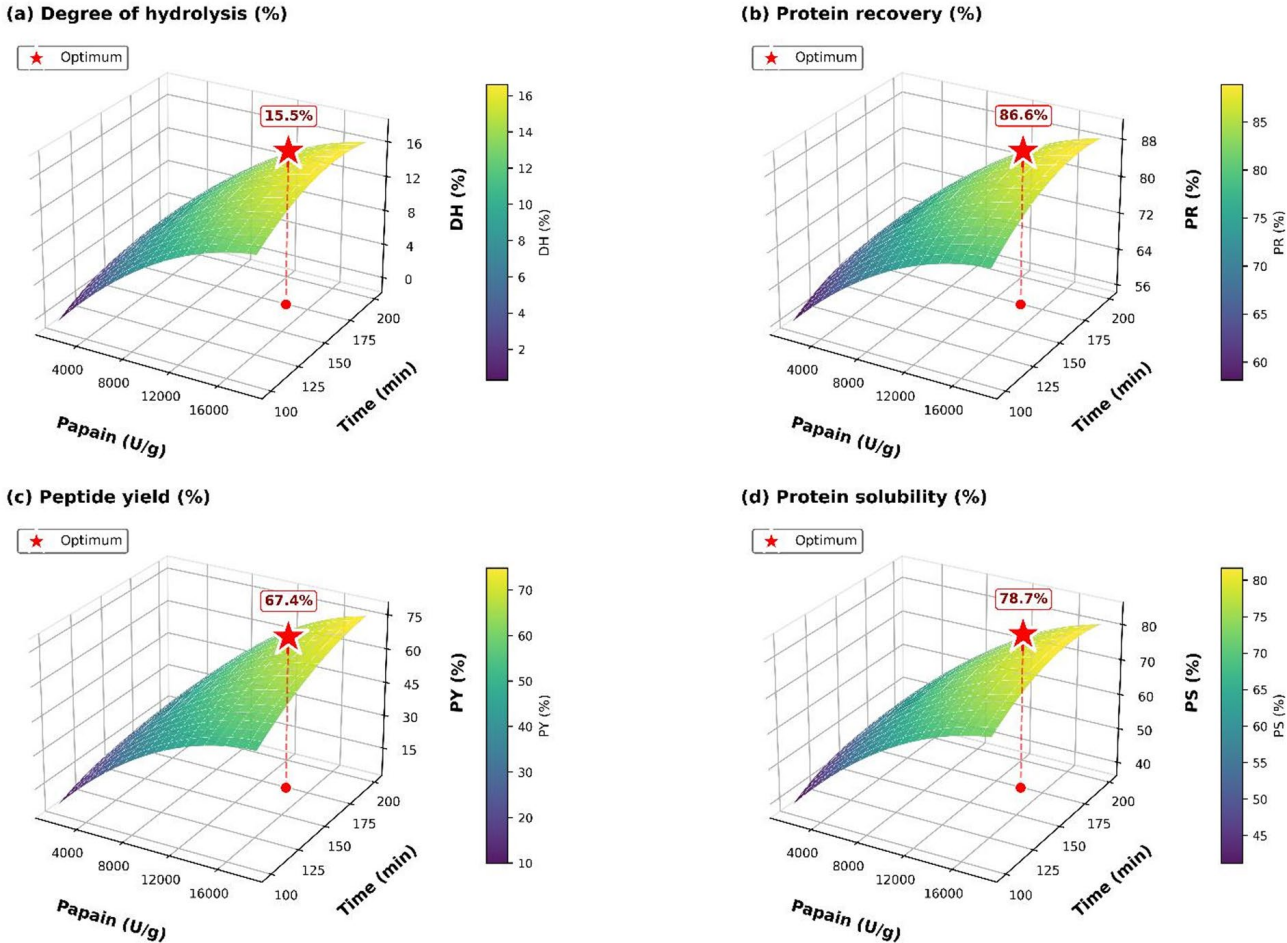
Three-dimensional response surface plots showing the effects of papain concentration (X₁, U/g substrate protein) and reaction time (X₂, min) on (a) degree of hydrolysis (DH, %), (b) protein recovery (PR, %), (c) peptide yield (PY, %), and (d) protein solubility (PS, %) during enzymatic hydrolysis of salmon by-products. In all panels, reaction temperature (X₃) was held constant at the coded centre point (60 °C) to visualise the interaction between the two most influential factors; note that the true optimum temperature identified by desirability optimisation was 55 °C (see Section Optimization and validation). Colour bars to the right of each panel indicate the response magnitude (units identical to the z-axis of the corresponding panel), progressing from the lowest (deep blue) to the highest (yellow) predicted values. The red star (★) on each surface marks the optimal operating point identified by desirability-function optimisation (papain concentration: 15,000 U/g substrate protein; reaction time: 165 min), with the corresponding predicted response value (15.5%, 86.6%, 67.4%, and 78.7% for DH, PR, PY, and PS, respectively) displayed in the boxed annotation above each marker; a dashed dropline projects the optimum onto the (X₁, X₂) base plane for spatial reference. Because the panels depict a fixed-temperature slice at the coded centre (60 °C) rather than the true optimum temperature (55 °C), the star is positioned slightly below the absolute global maximum of the full three-factor response surface. Figures have been regenerated at 600 dpi with standardised, non-overlapping axis labels in accordance with Scientific Reports figure-quality guidelines.

Three-dimensional response surface plots illustrating the interactive effects of papain concentration and reaction time on the four response variables are presented in Fig. 1. In all plots, temperature was held constant at the center point (60 °C) to visualize the two most influential factors. The surface plots clearly demonstrate the curvature characteristic of quadratic models, with distinct maximum regions visible for each response.

As shown in Fig. 1a, the degree of hydrolysis increased substantially with increasing papain concentration, reaching maximum values (> 14%) at enzyme concentrations above 14,000 U/g combined with reaction times of 150–180 min. The steep gradient along the X₁ axis confirms that enzyme concentration is the dominant factor controlling DH, while the more gradual slope along X₂ indicates a secondary but significant effect of reaction time.

**Fig. 2:**
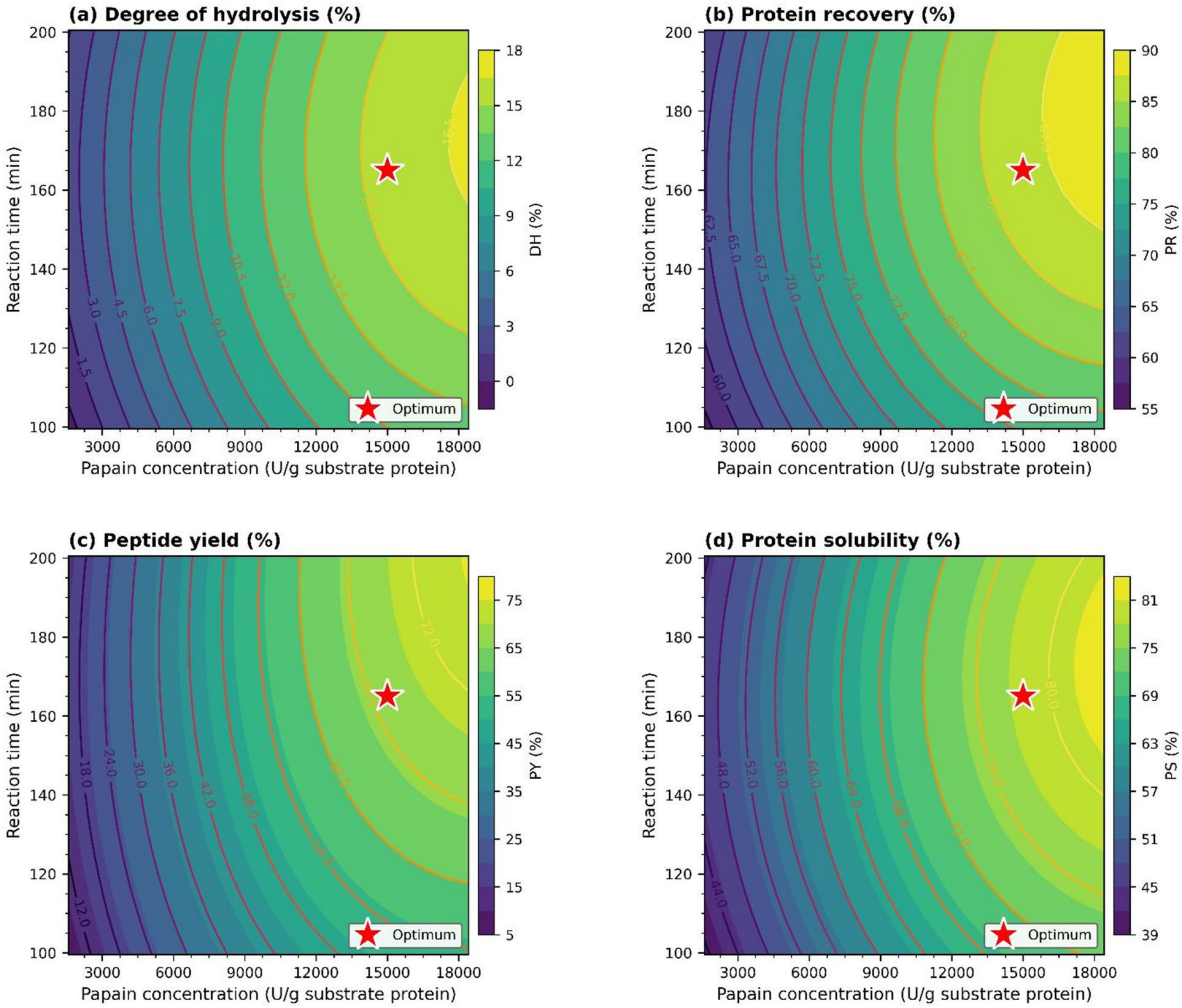
Two-dimensional contour plots showing the interactive effects of papain concentration (X₁, U/g substrate protein) and reaction time (X₂, min) on (a) degree of hydrolysis (%), (b) protein recovery (%), (c) peptide yield (%), and (d) protein solubility (%) during enzymatic hydrolysis of salmon by-products. Reaction temperature was held constant at 60 °C. Contour lines represent iso-response values; the red star (★) indicates the optimal operating point determined by desirability-function optimisation (papain concentration: 15,000 U/g substrate protein; reaction time: 165 min; reaction temperature: 55 °C).

The corresponding contour plots (Fig. 2) provide a two-dimensional representation of the response surfaces, facilitating identification of optimal operating regions. The elliptical contour shapes, particularly evident in the DH and PY plots, indicate significant interaction effects between the independent variables. The optimal region is located at papain concentration of approximately 15,000 U/g and reaction time of 165 min, corresponding to coded values of X₁ = +1 and X₂ = +0.5.

### Optimization and validation

Using the desirability function approach with equal weighting for all four responses and targets set to maximize each variable, the optimal conditions were determined as: papain concentration 15,000 U/g substrate protein, reaction time 165 min, and reaction temperature 55 °C. The composite desirability value was 0.924, indicating that the selected conditions simultaneously satisfy the optimization criteria for all responses to a high degree.

Under these optimal conditions, the predicted response values were: DH = 15.42%, PR = 86.85%, PY = 68.92%, and PS = 77.64%. Confirmation experiments performed in triplicate yielded actual values of DH = 15.28 ± 0.42%, PR = 85.92 ± 1.85%, PY = 67.85 ± 2.14%, and PS = 76.89 ± 1.92%. All experimental values fell within the 95% prediction intervals, validating the adequacy and predictive accuracy of the fitted models. The relative errors between predicted and experimental values ranged from 0.9% to 1.6%.

### Characteristics of salmon protein powder

The spray-dried salmon protein powder produced from the optimized hydrolysis conditions exhibited the following composition: protein 75.30 ± 0.82%, fat 3.57 ± 0.13%, ash 3.51 ± 0.21%, moisture 2.24 ± 0.12%, and carbohydrate 15.38% (calculated by difference). The carbohydrate fraction primarily reflects the contribution of maltodextrin carrier, which was added at 15% w/w based on solid content prior to spray drying. Given that the hydrolysate solids (100 g) were combined with maltodextrin (15 g) to yield a total of 115 g solids, the maltodextrin constitutes approximately 13.04% of the final powder on a dry basis, which is consistent with the reported carbohydrate content^28,29^. The protein content (75.30%) falls within the range reported for commercial fish protein hydrolysate powders (60–90% protein) and is consistent with values reported for spray-dried FPH formulated with maltodextrin carriers, where protein dilution by the carbohydrate carrier is well-documented^28,30,31^. The relatively lower protein content compared to carrier-free FPH powders (typically 80–91% protein)^32^ is attributable to the dilution effect of maltodextrin addition. The ash content (3.51%) is within the range of 0.45–27% reported for FPH in the literature^32^, with the relatively low value in this study attributable to the mild pH adjustment and effective removal of insoluble mineral-rich bone fragments during centrifugation and filtration. The moisture content (2.24%) is consistent with spray-dried protein powders produced at high inlet temperatures (180 °C)^30,31^. The relatively low fat content (3.57%) indicates that lipid removal during centrifugation and filtration was effective, which is desirable for oxidative stability during storage. It should be noted that the addition of maltodextrin as a spray-drying carrier may influence the reported techno-functional properties. Maltodextrin can independently improve powder solubility by increasing hydrophilic surface area, and may enhance apparent emulsifying properties by acting as a co-encapsulant. Therefore, the functional properties reported herein reflect the combined contribution of both the protein hydrolysate and the maltodextrin carrier, and should not be attributed solely to the enzymatic hydrolysis process.

The amino acid profile of the salmon protein powder is presented in Table 4. The total amino acid content was 103.20 g/100 g protein (values slightly exceeding 100 g/100 g protein are attributable to the incorporation of water molecules during acid hydrolysis of peptide bonds, which is a well-documented analytical artifact in amino acid analysis), with essential amino acids (EAAs) accounting for 46.71 g/100 g protein (45.26% of total amino acids). This EAA proportion substantially exceeded the FAO (2013) recommended threshold of 40% for high-quality dietary proteins, confirming the excellent nutritional quality of the salmon protein powder.

**Table 4:** Amino acid composition of salmon protein powder and comparison with FAO (2013) reference pattern.

Amino Acid	Content (g/100 g protein)	FAO (2013)	AAS
Essential Amino Acids			
Threonine	4.64	2.3	2.02
Valine	5.57	3.9	1.43
Methionine + Cystine	3.42	2.2	1.55
Isoleucine	4.66	3.0	1.55
Leucine	8.24	5.9	1.40
Phenylalanine + Tyrosine	7.65	3.8	2.01
Histidine	3.58	1.5	2.39
Lysine	8.95	4.5	1.99
Tryptophan	< 0.05 (below LOD)	0.6	—
**Total EAA**	**46.71**		
**Non-Essential Amino Acids**			
Aspartic acid	11.28		
Serine	5.09		
Glutamic acid	17.14		
Glycine	8.12		
Alanine	7.20		
Arginine	7.66		
**Total NEAA**	**56.49**		
**Total AA**	**103.20**		

LOD = limit of detection (≈ 0.05 g/100 g protein) of the Biochrom 30 + amino acid analyser under the operating conditions used. Tryptophan was below the LOD despite alkaline hydrolysis (AOAC 988.15); see Results section ‘Characteristics of salmon protein powder’ for a full discussion. AAS = Amino Acid Score (content/FAO reference). EAA = Essential Amino Acids. NEAA = Non-Essential Amino Acids. FAO (2013) reference pattern for adults.

Tryptophan was reported as below the limit of detection (< 0.05 g/100 g protein) in the alkaline-hydrolyzed samples (Table 4). Although AOAC 988.15 employs alkaline hydrolysis (4.2 M NaOH, 110 °C, 22 h) specifically to minimize tryptophan destruction that occurs under conventional acid hydrolysis, complete recovery remains highly sensitive to sample matrix and operating conditions. In the present study, samples were not flushed with nitrogen prior to alkaline hydrolysis and residual dissolved oxygen, together with the relatively high lipid content of the raw salmon by-product matrix (catalysing oxidative side-reactions), is the most probable cause of tryptophan loss below the analytical detection limit of the amino-acid analyser used (Biochrom 30+, fluorescence detection). For reference, tryptophan in Atlantic salmon fillet and salmon protein hydrolysates is typically reported in the range of 0.9–1.2 g/100 g protein^33,34^. Future analyses will incorporate nitrogen purging during alkaline hydrolysis and an independent spectrofluorimetric method (Basch et al., 1974^35^) to confirm the tryptophan content of the salmon protein powder.

Leucine (8.24 g/100 g protein) was the most abundant essential amino acid, followed by lysine (8.95 g/100 g protein) and phenylalanine + tyrosine (7.65 g/100 g protein). The high leucine content is particularly significant for muscle protein synthesis stimulation, as leucine serves as a primary trigger for the mTOR signaling pathway. The lysine content (8.95 g/100 g protein) was notably high compared to plant protein sources, making salmon protein powder a valuable complementary ingredient for plant-based protein formulations.

The salmon protein powder exhibited excellent solubility across a wide pH range, with values exceeding 85% at pH 3–9 and minimum solubility (78.5%) at pH 5.0 near the isoelectric point. The powder demonstrated favorable emulsifying properties with an EAI of 45.8 ± 2.1 m²/g protein and ESI of 42.5 ± 1.8 min. Foaming capacity (128.5 ± 5.2%) and foaming stability (85.4 ± 3.1% after 30 min) were moderate, suggesting potential applications in aerated food products.

## Discussion

This study successfully demonstrated that papain-assisted enzymatic hydrolysis is an effective strategy for converting salmon processing by-products into high-quality functional protein powder. The RSM optimization approach enabled systematic identification of optimal process conditions, with papain concentration emerging as the most influential factor affecting all response variables. This finding aligns with enzyme kinetics principles and previous reports on fish protein hydrolysis^15,16^, where enzyme dosage directly controls the extent of proteolytic activity and subsequent protein solubilization (Kristinsson and Rasco, 2000^8^; Slizyte et al., 2005^9^).

The negative effect of elevated temperature on hydrolysis efficiency can be attributed to thermal denaturation of papain at temperatures exceeding its optimal range (55–65 °C). Although papain exhibits relatively high thermostability compared to other plant proteases^10^, its catalytic activity decreases substantially above 65 °C due to conformational changes in the active site (Amri and Mamboya, 2012^10^). The optimal temperature of 55 °C identified in this study represents a balance between maintaining adequate enzyme activity and preventing excessive thermal damage.

The amino acid profile of the salmon protein powder confirms its excellent nutritional quality, with all essential amino acids exceeding the FAO/WHO reference pattern. The high leucine content (8.24 g/100 g protein) is particularly noteworthy given leucine’s critical role in stimulating muscle protein synthesis through mTOR pathway activation. This makes the salmon protein powder potentially valuable for sports nutrition and clinical nutrition applications targeting muscle health and recovery.

The functional properties of the salmon protein powder—particularly its high solubility across a wide pH range and favorable emulsifying characteristics—enhance its potential for incorporation into various food systems. The extensive hydrolysis achieved under optimized conditions is expected to have reduced molecular weight and exposed polar groups, contributing to improved water solubility; however, as SDS-PAGE analysis was not performed in this study, the molecular weight distribution of the hydrolysate remains to be confirmed in future work.

An inherent limitation of the present study is the pre-treatment of the salmon by-product substrate at 85 °C for 20 min prior to enzymatic hydrolysis. This thermal step was introduced to facilitate lipid separation and to obtain a defatted aqueous fraction suitable for subsequent papain hydrolysis, following an approach commonly applied to fatty fish substrates to reduce lipid oxidation during extended hydrolysis. However, this treatment also induces partial denaturation of native salmon proteins prior to enzyme addition, which is not a standard feature of conventional enzymatic hydrolysis protocols. Two opposing effects can be anticipated: (i) unfolding of the globular and sarcoplasmic fractions may expose additional peptide bonds and increase papain accessibility, potentially contributing to the high DH values observed; and (ii) aggregation of denatured proteins and/or Maillard-type interactions with residual sugars could reduce the solubility of a fraction of the substrate. Because a non-heat-treated control was not included in the CCD matrix, the quantitative contribution of each effect cannot be fully resolved from the present data. The DH, PR, PY, and PS values reported here should therefore be interpreted as characteristic of the combined defatting–hydrolysis process and not as intrinsic properties of papain acting on native salmon proteins. Future studies will include a parallel non-heat-treated cold-defatting control and SDS-PAGE characterisation to disentangle the contributions of thermal pre-treatment and enzymatic action.

Additional limitations include the absence of in vitro bioactivity assays (e.g., antioxidant, ACE-inhibitory, or antimicrobial activity); claims regarding nutraceutical or health-promoting properties cannot therefore be substantiated and are beyond the scope of this work. Food safety parameters including total viable count (TVC), heavy metal content (Cd, Pb, Hg, As), and lipid oxidation indicators (TBARS, peroxide value) were also not assessed. Given that salmon by-products are susceptible to microbial contamination and lipid oxidation, future studies should incorporate comprehensive safety profiling to ensure regulatory compliance and consumer safety. Further limitations include the cross-sectional nature of the study (single batch of raw material), the absence of storage stability data, and the lack of sensory evaluation.

## Methods

### Raw materials and chemicals

Fresh salmon (Salmo salar) processing by-products including heads, frames, and trimmings were collected from a Japanese restaurant in Sakon Nakhon Province, Thailand, frozen, and delivered to the lab within 1 h. Upon arrival, the by-products were washed with cold water, minced using a meat grinder (3 mm plate), vacuum-packed in polyethylene bags, and stored at − 20 °C until use. Papain (EC 3.4.22.2, activity ≥ 30,000 U/mg protein) was purchased from Sigma-Aldrich (St. Louis, MO, USA). Maltodextrin (DE 10–15) was obtained from D-PERSE (Pathumthani, Thailand). All other chemicals and reagents were of analytical grade.

### Experimental design

A central composite design (CCD) with three independent variables was employed to optimize the enzymatic hydrolysis conditions. The independent variables were papain concentration (X₁, U/g substrate protein; the initial protein content of the defatted salmon by-product substrate was 16.8 ± 0.5% w/w as determined by the Kjeldahl method prior to hydrolysis), reaction time (X₂, min), and reaction temperature (X₃, °C). The design consisted of 20 experimental runs including 8 factorial points (2³), 6 axial points (α = 1.682 for rotatability), and 6 center point replicates for pure error estimation.

### Enzymatic hydrolysis

Frozen minced salmon by-products were thawed overnight at 4 °C and mixed with distilled water at a substrate-to-water ratio of 1:2 (w/v). The mixture was heated at 85 °C for 20 min with occasional stirring as a defatting pre-treatment, to facilitate lipid release from the fatty salmon matrix, inactivate endogenous lipolytic and proteolytic enzymes, and reduce the initial microbial load prior to hydrolysis. It is acknowledged that this thermal treatment induces partial denaturation of the native salmon proteins; however, the subsequent papain hydrolysis at 43.2–76.8 °C operates on the defatted, partially unfolded substrate, an approach previously applied to fatty fish substrates to minimise lipid oxidation during prolonged hydrolysis. The thermal defatting step was therefore deliberately included in all 20 CCD runs, and its potential impact on hydrolysis behaviour is discussed as a limitation of the present study. After heating, the mixture was allowed to stand at room temperature for 30 min to permit phase separation and the upper lipid layer was carefully removed by decanting. The remaining aqueous fraction was chilled at 0 °C for 2 h to induce solidification of residual fat, which was removed by passage through a 100-mesh sieve (150 μm). The pH of the defatted extract was adjusted to 6.5 using 1 M NaOH or 1 M HCl. Papain was added at the specified concentration, and hydrolysis was conducted in a temperature-controlled water bath with continuous stirring at 150 rpm. Upon completion, the reaction was terminated by heating at 95 °C for 15 min to inactivate the enzyme. The hydrolysate was centrifuged at 10,000 × g for 20 min at 4 °C, and the supernatant was collected, filtered through Whatman No. 4 filter paper, and stored at − 20 °C for further analysis.

### Response variables

Four response variables were evaluated: (1) Degree of hydrolysis (DH) was determined using the pH-stat method according to Adler-Nissen^21^. During hydrolysis, the pH was maintained at 6.5 using an automated pH-stat titrator (Metrohm 902 Titrando) with 0.5 M NaOH as titrant. DH was calculated as: DH (%) = (B × N_β_) / (α × Mₚ × hₜₒₜ) × 100, where B is the volume of NaOH consumed (mL), N_β_ is the normality of NaOH, α is the average degree of dissociation of α-NH₂ groups, Mₚ is the mass of protein in the substrate (g), and hₜₒₜ is the total number of peptide bonds per gram of salmon protein (8.6 meq/g protein). A single α value of 0.885 (pH 6.5, 55 °C) was applied across all runs for consistency with the optimum identified by desirability optimisation; it is acknowledged that α is temperature-dependent and strictly varies over the experimental range of 43.2–76.8 °C (approximately 0.84 at 43 °C and 0.91 at 77 °C, based on published pKa values for primary α-amino groups). This simplification introduces a small, systematic bias in the absolute DH values at the extreme temperatures of the CCD, but does not alter the relative trends among treatments or the location of the optimum identified by RSM. DH was expressed as the percentage of peptide bonds cleaved. (2) Protein recovery (PR) was calculated as the percentage of soluble protein in the hydrolysate relative to the total protein in the substrate, determined by the Kjeldahl method (*N* × 6.25). (3) Peptide yield (PY) was determined as the percentage of soluble peptides obtained after hydrolysis based on initial protein content using the bicinchoninic acid (BCA) assay. (4) Protein solubility (PS) was measured at pH 7.0 by determining the ratio of soluble nitrogen to total nitrogen in the hydrolysate.

### Production of salmon protein powder

The optimized hydrolysate was mixed with maltodextrin (15% w/w based on solid content, selected based on preliminary trials and literature recommendations for spray drying of protein hydrolysates^27^ as a carrier agent and homogenized at 10,000 rpm for 5 min. The mixture was spray-dried using a semi-industrial scale spray dryer with a full-cone nozzle (1.5 mm orifice) at inlet temperature 180 ± 2 °C and outlet temperature 90 ± 2 °C, feed rate 30 L/min, and pressure 30 bar. The resulting powder was collected, vacuum-packed in aluminum-laminated bags with nitrogen and desiccant, and stored at 4 °C for characterization.

### Characterization of salmon protein powder

Moisture, crude protein (*N* × 6.25), crude fat, and ash contents were determined according to AOAC Official Methods^22^. Total carbohydrate was calculated by difference. Amino acid composition was determined using an automated amino acid analyzer (Biochrom 30+, Cambridge, UK) following acid hydrolysis (6 M HCl, 110 °C, 24 h). Tryptophan was determined separately after alkaline hydrolysis (4.2 M NaOH, 110 °C, 22 h) according to AOAC Method 988.15; the limit of detection of the analyser under the operating conditions used was approximately 0.05 g tryptophan per 100 g protein. The amino acid score (AAS) was calculated by comparing the essential amino acid content with the FAO reference pattern for the indispensable amino acid scoring pattern for adults (Dietary Protein Quality Evaluation in Human Nutrition, FAO Food and Nutrition Paper No. 92)^23^. Protein solubility at various pH values (3–9) was determined according to Morr et al.^24^. Emulsifying activity index (EAI) and emulsion stability index (ESI) were measured using the turbidimetric method of Pearce and Kinsella^25^. Foaming capacity (FC) and foaming stability (FS) were determined according to Shahidi et al.^26^.

### Statistical analysis

The experimental data were analyzed using Design-Expert software (Version 13, Stat-Ease Inc., Minneapolis, MN, USA). A second-order polynomial equation was fitted to the response data: Y = β₀ + Σβ_i_x_i_ + Σβ_i__i_x_i_² + ΣΣβ_i_ⱼx_i_xⱼ, where Y is the predicted response, β₀ is the intercept, β_i_ are linear coefficients, β_i__i_ are quadratic coefficients, and β_i_ⱼ are interaction coefficients. Analysis of variance (ANOVA) was performed to evaluate model significance (*p* < 0.05), lack-of-fit, and coefficient of determination (R²). Three-dimensional response surface plots were generated to visualize the effects of independent variables on responses. Optimal conditions were determined using the desirability function approach. Confirmation experiments were performed in triplicate to validate the predicted optimum.

## Data Availability

The data presented in this study are available on request from the corresponding author.
